# Risk and Resilience Pathways, Community Adversity, Decision-making, and Alcohol Use Among Appalachian Adolescents: Protocol for the Longitudinal Young Mountaineer Health Study Cohort

**DOI:** 10.2196/40451

**Published:** 2022-08-05

**Authors:** Alfgeir L Kristjansson, Annette M Santilli, Rosalina Mills, Hannah M Layman, Megan L Smith, Michael J Mann, James MacKillop, Jack E James, Christa L Lilly, Steven M Kogan

**Affiliations:** 1 Department of Social and Behavioral Sciences School of Public Health West Virginia University Morgantown, WV United States; 2 School of Public Health West Virginia University Morgantown, WV United States; 3 School of Public and Population Health Boise State University Boise, ID United States; 4 Peter Boris Centre for Addictions Research McMaster University Hamilton, ON Canada; 5 St Joseph’s Healthcare Hamilton Hamilton, ON Canada; 6 Department of Psychology Reykjavik University Reykjavik Iceland; 7 Department of Epidemiology and Biostatistics School of Public Health West Virginia University Morgantown, WV United States; 8 Department of Human Development and Family Science University of Georgia Athens, GA United States

**Keywords:** adolescence, middle school, Appalachia, caffeine, alcohol use, Young Mountaineer Health Study, prevention

## Abstract

**Background:**

Alcohol use impairs psychosocial and neurocognitive development and increases the vulnerability of youth to academic failure, substance use disorders, and other mental health problems. The early onset of alcohol use in adolescents is of particular concern, forecasting substance abuse in later adolescence and adulthood. To date, evidence suggests that youth in rural areas are especially vulnerable to contextual and community factors that contribute to the early onset of alcohol use.

**Objective:**

The objective of the Young Mountaineer Health Study is to investigate the influence of contextual and health behavior variables on the early onset of alcohol use among middle school–aged youth in resource-poor Appalachian rural communities.

**Methods:**

This is a program of prospective cohort studies of approximately 2200 middle school youth from a range of 20 rural, small town, and small city (population <30,000) public schools in West Virginia. Students are participating in 6 waves of data collection (2 per year) over the course of middle school (sixth to eighth grades; fall and spring) from 2020 to 2023. On the basis of an organizational arrangement, which includes a team of local data collection leaders, supervising contact agents in schools, and an *honest broker* system to deidentify data linked via school IDs, we are able to collect novel forms of data (self-reported data, teacher-reported data, census-linked area data, and archival school records) while ensuring high rates of participation by a large majority of youth in each participating school.

**Results:**

In the spring of 2021, 3 waves of student survey data, 2 waves of data from teachers, and a selection of archival school records were collected. Student survey wave 1 comprised 1349 (response rate 80.7%) participants, wave 2 comprised 1649 (response rate 87%) participants, and wave 3 comprised 1909 (response rate 83.1%) participants. The COVID-19 pandemic has had a negative impact on the sampling frame size, resulting in a reduced number of eligible students, particularly during the fall of 2020. Nevertheless, our team structure and incentive system have proven vitally important in mitigating the potentially far greater negative impact of the pandemic on our data collection processes.

**Conclusions:**

The Young Mountaineer Health Study will use a large data set to test pathways linking rural community disadvantage to alcohol misuse among early adolescents. Furthermore, the program will test hypotheses regarding contextual factors (eg, parenting practices and neighborhood collective efficacy) that protect youth from community disadvantage and explore alcohol antecedents in the onset of nicotine, marijuana, and other drug use. Data collection efforts have been successful despite interruptions caused by the COVID-19 pandemic in 2020 and 2021.

**International Registered Report Identifier (IRRID):**

DERR1-10.2196/40451

## Introduction

Underage youth drink 11% of the total alcohol consumed in the United States [1]. The personal and social consequences of underage drinking are staggering; each year, approximately 5000 deaths of minors can be attributed to alcohol use [2]. Alcohol use impairs psychosocial and neurocognitive development and increases the vulnerability of youth to drug abuse, academic failure, high-risk sexual behavior, and mental health problems [1]. The onset of alcohol use during middle school years is particularly problematic, forecasting problems with alcohol and other substances during adolescence [3] and chronic substance use problems in adulthood [4].

The Young Mountaineer Health Study (YMHS) is a prospective school-based investigation of the development of alcohol use vulnerability among Appalachian youth in West Virginia (WV). WV, the only US state located entirely within the Appalachian region, has among the highest poverty rates in the United States [5]. The region’s coal mining areas, in particular, evince low economic diversification, low employment in professional services, and low rates of educational attainment, which have contributed to pervasive health disparities [6,7] and epidemic levels of opioid abuse and mortality [8]. Historically, residing in rural communities in general, including Appalachian communities, has protected youth from many of the risk factors for early onset alcohol use encountered in urban settings. However, recent epidemiological research underscores the prevalence of alcohol use in adolescents and adult alcoholism in the resource-poor Appalachian environments [9-12]. Of particular concern, early onset alcohol use among rural Appalachian youth places them at risk for future substance use, including the nonmedical use of prescription drugs [13-15].

Current etiological models of the development of alcohol use vulnerability in the Appalachian region emphasize the proliferation of risk factors in resource-poor communities [16]. Poverty, isolation, and unemployment affect neighborhood, school, and family contexts. This, in turn, undermines academic engagement and increases the likelihood of alcohol use and affiliation with peers who support it [16]. Appalachian youth may be exposed to rearing environments that are generally more alcohol-friendly than those experienced by their urban peers [17]. Alcohol is readily available as surveillance of service to minors in alcohol retail outlets is less stringent in rural areas [2]. Investigations of unique stressors in Appalachian and other rural contexts have yielded considerable progress, informing an initial generation of prevention programs [18] and policy-related initiatives designed to reduce exposure of youth to key contextual risk factors. Nevertheless, emerging research on the influence of lifestyle factors on alcohol use vulnerability suggests that these models may omit important risk mechanisms, undermining the effectiveness of the programs they inform. In particular, this study considers the influence of caffeine use and sleep health in the etiology of early onset alcohol use.

**Figure 1:**
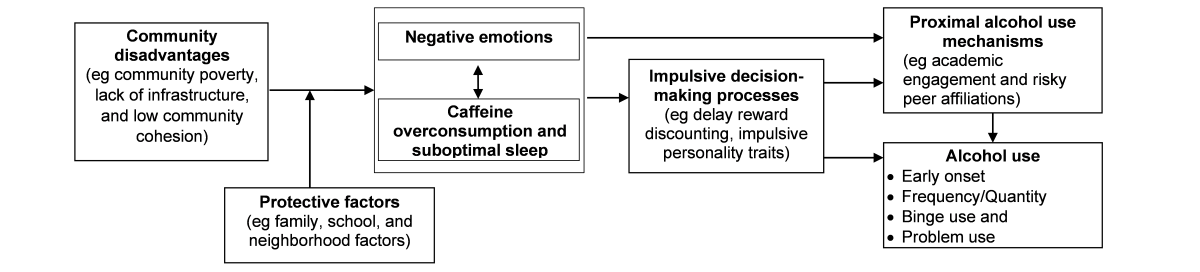
Risk and protective pathways linking community disadvantage to early adolescent alcohol use.

Figure 1 presents the conceptual model guiding the study. It specifies the risk and protective pathways linking residence in disadvantaged community environments to the development of alcohol use vulnerability among Appalachian youth. As shown in Figure 1, disadvantaged community environments promote negative affect and problems concerning the regulation of emotions. Studies have documented elevated levels of depressive symptoms, anger, and anxiety in resource-poor Appalachian communities [19]. Similar links with emotion regulation, including emotional awareness and the ability to modulate distressing emotions, have emerged [20,21]. We hypothesize that negative emotionality, caffeine use, and sleep problems form a system of mutually reinforcing behaviors. The stimulant effects of caffeine tend to be short lived and followed by increased irritability and reductions in the quality [22] and quantity [23] of adolescents’ sleep. Youth often increase their caffeine consumption to combat irritability and drowsiness, thus forming a cycle of reinforcement. Sleep may also be disrupted as a consequence of neighborhood disadvantage. Specifically, youth from resource-poor communities report more disruptions in sleep and greater negative consequences in school achievement because of sleep disruptions [23]. Thus, youth from resource-poor Appalachian environments may be expected to report heightened problems with negative emotions, caffeine use, and sleep problems.

We hypothesize that problems with sleep, caffeine, and negative emotionality carry forward to affect alcohol use through proximal alcohol use mechanisms, including youth self-regulation, youth academic engagement, and youths’ affiliations with risk-taking peers. Negative affectivity promotes impulsive decision-making, which reduces the extent to which youth consider the consequences of alcohol use when faced with an opportunity to drink [24]. Cycles of irritability and negative emotionality reinforced by caffeine use and sleep-related problems are expected to undermine academic achievement and school engagement. Reductions in academic engagement in middle school are robust antecedents of early onset alcohol use and alcohol misuse in high school [25]. Youth who experience negative emotions and impulsive decision-making, as well as those with low attachment to school, tend to select friends with similar characteristics who are likely to provide opportunities and reinforcement for using substances [26].

Despite exposure to community disadvantage, most young people avoid problems with alcohol use. Resilience models focus on the sources of this protection. As shown in Figure 1, we hypothesize that protective factors in the family, school, and local community contexts will attenuate the influence of community disadvantage on caffeine use and poor sleep, negative emotionality, and decision-making. The proximity, reliability, and durability of family relationships represent the focal points of life in Appalachian communities [27]. Multiple studies in other populations attest to the power of effective parenting practices to shield youth from the effects of poverty [28]. Protective parenting styles include high levels of youth monitoring, consistent discipline and family routines, and the expression of support and affection [29,30]. In studies of minority youth living in disadvantaged rural communities, these parenting styles attenuated the influence of poverty on a range of outcomes, including internalizing and externalizing problems and affiliation with deviant peers [31,32]. For youth residing in challenging communities, parental regulation and family routines foster self-regulation and emotion regulation, thus allowing them to stay focused on school and avoid risky peers and dangers in the community [33]. However, these factors have not yet been investigated in Appalachia.

For youth residing in disadvantaged communities, effective schools can be a refuge that supports social, emotional, and cognitive development [34]. In many central Appalachian communities, schools play a vital role in providing students with a life structure, a consistent supply of meals, and access to physical resources in a secure environment, which are not commonly available to students at home [35]. Students are more likely to engage in healthy behaviors and succeed academically when they feel strong connections with school [36]. The protective aspects of effective schools include classroom dynamics (high demand, high warmth teachers, and classroom consistency), school-wide climate (safety and positive affect), and opportunities to engage with extracurricular activities [37]. In Appalachia, geographic dispersion and a lack of municipal areas and infrastructure can create a strong sense of interdependence, trust, and cohesion among local community members [27]. Community systems have the potential to affect adolescent development, particularly when neighbors intervene to assist vulnerable families struggling with economic distress or substance-related problems [38]. Studies of disadvantaged communities have underscored the influence of collective efficacy in shielding adolescent development [39]. Collective efficacy includes collective socialization, neighbors taking responsibility for the care and monitoring of others’ children, social cohesion, and a sense of trust in local community relationships. To our knowledge, prospective studies of the buffering influence of these factors on youth development in Appalachian communities have not been conducted.

In a sample of early adolescents in Appalachia, our three specific aims are as follows.

- To test the pathways linking community disadvantage to alcohol misuse; we expect community disadvantage to forecast slower growth in decision-making processes directly and indirectly via negative emotionality, which is reinforced by the use of caffeine and attendant problems with sleep; in turn, we expect negative emotionality and decision-making trajectories to forecast alcohol misuse directly and indirectly via proximal vulnerability factors (affiliation with risky peers and academic disengagement)
- To test hypotheses regarding contextual factors (eg, parenting practices and neighborhood collective efficacy) that protect youth from community disadvantage; we expect contextual protective factors to attenuate the influence of community disadvantage on negative emotionality, caffeine use and sleep problems, and decision-making trajectories
- To explore the aforementioned alcohol antecedents in the onset of nicotine, marijuana, and other drug use

## Methods

### Methodological Overview, Design Considerations, and the “Honest Broker” System

We proposed to test the hypotheses presented in Figure 1 in a prospective study of middle school youth from a range of rural, small town, and small city (population <30,000) public schools (N=20) in WV. Students will participate in 6 waves of data collection (2 per year) over the course of middle school (sixth to eighth grade). Drawing on our school partnerships and “honest broker” system described in the following paragraphs, we will be able to collect multimethod data (self-reported data, teacher-reported data, school records, and census-linked area data) and ensure high rates of participation by most youth in each school. This design allows us to investigate growth and changes in alcohol use vulnerability factors across the transition to adolescence. This study was funded by the National Institute on Alcohol Abuse and Alcoholism, with an official start date of October 9, 2019 (Multimedia Appendix 1).

The YMHS uses a novel data collection system to gather prospective multimethod assessments and ensure high rates of participation by most youth in each school. At the same time, it should be acknowledged that prospective multimethod research through school systems poses unique challenges. The most significant involves obtaining sufficient percentages of youth whose parents consent to their participation. In resource-poor rural areas, the youth from whom data collection is most urgent may have disordered living situations that may prevent the obtaining of parental or caregiver consent for many young individuals. Over the past 8 years, our team has collaborated with school officials, parents, and the institutional review board (IRB) of the university to develop a system that addresses the needs and rights of youth and parents; the responsibilities of researchers, IRBs, and school officials; and the critical need for data on youth exposed to Appalachian poverty. In accordance with the Family Education Rights and Privacy Act guidelines, schools may collect data for evaluation purposes, and these data may be shared with researchers if deidentified to the investigative team. Thus, we developed a system of school contact agents and school-based officials who collect self-reported and school record data, which are deidentified before being accessed by study investigators and analysts via an “honest broker” system. The honest broker creates a research ID for each individual respondent and secures a key linking school ID numbers to the nonidentifiable research IDs. This deidentifies the data to the research team while allowing the collection of survey data, census-linked zip code information, and school record–based data for research purposes. Parents passively consent (by way of an opt-out letter sent to the home), and the youth provide informed assent for their participation. This facilitates the collection of prospective data from most students for research purposes. Importantly, researchers provide reports back to the schools, which allows them to identify the risk factors and needs for planning interventions and seeking funds for extracurricular and intervention-based activities within the school system. We pilot-tested this system by collecting 3 waves of data on an annual basis (2015-2018) from 16 middle schools in WV [40,41].

### Recruitment Strategy

The baseline sampling frame included all sixth-grade students from 20 middle schools in 5 counties in WV (approximately 2200). Securing the recruitment of participants required the involvement of many parties. Given our preliminary data and previous data collection efforts in WV [41,42], it appeared logical to seek continued collaboration with public schools. The first step in securing their collaboration was to pursue approval from the highest level of educational authority at the local level, which, in WV, are the County Superintendents and the Boards of Education (BoEs). Counties were selected based on prior collaborations and their potential to contribute to a maximally diverse sample. Schools within the counties were included from communities designated as remote rural, distant rural, fringe rural, distant town, fringe town, small suburb, and small city [43]. To begin communication and outreach to counties and schools, the investigative team developed a Memorandum of Understanding (MoU), which described the project, its aims, school involvement, and school-based incentives. The MoU was then submitted and reviewed by the superintendents or their designees, which was subsequently followed by in-person meetings between the principal investigators and superintendents and a mutual signing of the MoUs. In some cases, this process required several meetings and involved other associated parties such as members of the BoEs, school principals, or their designees. The next step in this process was to engage in individual meetings with individual school principals. During this process, one county decided not to participate in the project, and another with a similar demographic profile was recruited in their place. Once principals had approved their schools to participate in the study, they were asked to nominate a supervising contact agent (SCA) for their school. The SCA is an individual in each school with whom the investigative team collaborated most closely regarding all aspects of the data collection, such as finding suitable dates and times and location of survey administration delivery. The SCA is often the principal, assistant principal, or guidance counselor. Acknowledging that schools are busy places with a different mission than that of collecting data for researchers, having access to a single individual in each school has proven very important for data collection quality and the securing of high response rates.

### Organizational Chart

**Figure 2:**
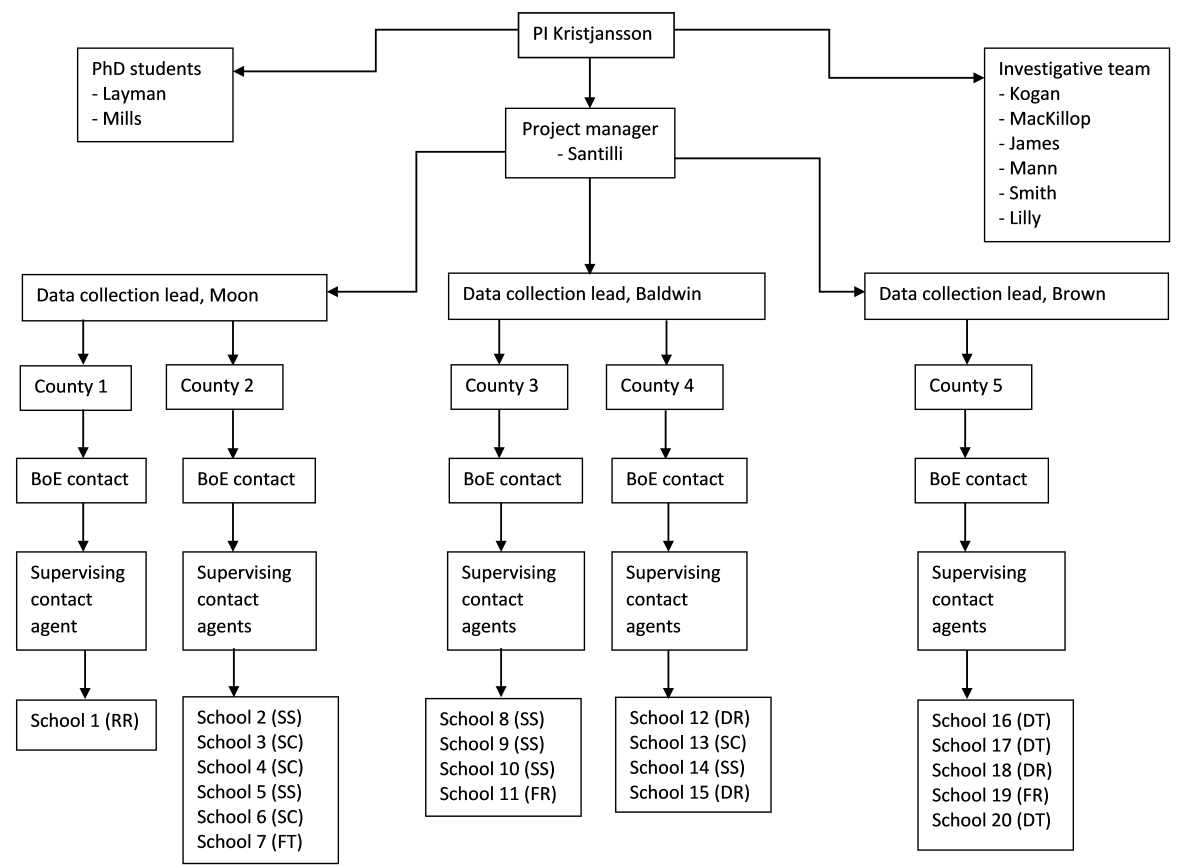
Young Mountaineer Health Study organizational chart. BoE: Board of Education; DR: distant rural (approximately 10 miles from town); DT: distant town (approximately 20 miles from city); FR: fringe rural (approximately 5 miles from town); FT: fringe town (approximately 5 miles from city; small suburban area within a city); PI: principal investigator; RR: remote rural (>25 miles from town); SC: small city.

Figure 2 presents the organizational chart for the project, including the investigative team, students, and staff. Once funding was secured, we proceeded to complete the organization of the planned data collection procedures, which included the training and hiring of relevant personnel. A full-time project director and 3 data collection leaders were hired. In line with the principles of community-engaged research [44], an important prerequisite for hiring data collection leaders was their understanding and experience with local schools. The 3 data collection leaders were all either retired teachers or close to retirement teachers from the local communities of the schools. The fact that our data collection leaders are experienced teachers from the localities under investigation has proved vitally important in the mitigation of various challenges that have come up during data collection, including issues associated with the COVID-19 pandemic.

### Data Collection Methods and Measures

Data collection protocols include a student report survey, a teacher report survey, and archival data from the BoEs and schools. The student survey includes close to 300 variables and requires 1 full class period (approximately 45 minutes) to complete. Teacher survey data were collected from teachers for each participant. It includes 37 questions on three constructs: self-control [45], peer affiliations and social acceptance by peers [46], and problem behavior [47]. Finally, archival data are collected from the BoEs and schools and include zip codes, grades, free or reduced lunch status, and disciplinary actions. These data are collected routinely by schools, and zip codes are linked to area data from the American Community Survey to provide contextual information on community affluence.

Table 1 lists the constructs included in the data collection, their source, and relevant references.

**Table 1:** Measurement concepts in the Young Mountaineer Health Study.

Construct	Source	Reference
Family affluence	Student survey	Health Behavior in School-aged Children study [48]
School climate	Student survey	Resnick et al [36]
School as a protective factor	Student survey	Mann, MJ, unpublished data, May 2022
Peer delinquency	Student survey	Heimer and Matsueda [49]
Peer ATOD^a^ use	Student survey	Kristjansson et al [50]
Perceived peer respect for ATOD use	Student survey	Kristjansson et al [50]
Perceived parental reactions to ATOD use	Student survey	Kristjansson et al [50]
Daytime sleepiness	Student survey	Meltzer et al [51]
Sleep disturbance	Student survey	Meltzer et al [51]
Caffeine consumption	Student survey	James et al [52]
ATOD use	Student survey	Monitoring the Future, ESPAD^b^ [53,54]
Anxiety	Student survey	Derogatis et al [55]
Depressed mood	Student survey	Derogatis et al [55]
Anger	Student survey	Derogatis et al [55]
Conduct disorder	Student survey	Lewinsohn et al [56]
Family management	Student survey	Communities that Care [57]
Parental monitoring	Student survey	Sigfúsdóttir et al [58]
Social capital	Student survey	Sigfúsdóttir et al [58]
Dysfunctional parenting	Student survey	Arnold et al [59]
Caregiver support	Student survey	Schaefer [60]
Impulsive personality traits	Student survey	Watts et al [61]
Delayed reward discounting	Student survey	Kirby et al [62]
Community safety	Student survey	Echeverria et al [63]
Perceived access to drugs	Student survey	Cochran et al [64]
Neighborhood ties	Student survey	Bernburg et al [65]
Collective efficacy	Student survey	Sampson et al [39]
Organized leisure time activities	Student survey	Kristjansson et al [66]
Pubertal development	Student survey	Carskadon and Acebo [67]
Life satisfaction	Student survey	Seligson et al [68]
COVID-19 exposure and impact	Student survey	N/A^c^
Problem behavior	Teacher survey	Piper et al [47]
Peer affiliations and social acceptance	Teacher survey	Dishion et al [46]
Self-control	Teacher survey	Humphrey [45]
Zip codes	Schools/BoE^d^	N/A
Census tracks	Am Com survey	N/A
Grades	Schools/BoE	N/A
Free or reduced lunch status	Schools/BoE	N/A
Disciplinary actions	Schools/BoE	N/A

^a^ATOD: alcohol, tobacco, and other drugs.

^b^ESPAD: European School Survey Project on Alcohol and Other Drugs.

^c^N/A: not applicable.

^d^BoE: Board of Education.

### Pilot Study

During the summer of 2020, the student survey was pilot-tested with a convenience sample of 15 youths (n=6, 40% boys; n=8, 53% girls; and n=1, 7% gender nonconforming) aged 11 to 12 years who lived in areas in WV that were not part of the YMHS study. The purpose of the pilot study was to assess the students’ understanding of the questions and response categories and to determine the length of time required to complete the survey. Furthermore, a selection of teachers and SCAs reviewed both the study questionnaire and teacher survey. Comments from all parties were combined into a file and used to make minor adjustments to the wording and order of questions. Both the student and teacher surveys were deemed to be of appropriate length for completion in 1 full class period, and no pilot participants reported significant problems in understanding the survey items.

### Data Collection Procedures

All parents or caregivers received an introductory letter 2 weeks before data collection, which described the study and permitted parents to opt out their children from participating. The letters were sent via regular mail, take-home mail with students, or email via school listservs. Many schools have used multiple ways of contacting parents or caregivers about study involvement. The youth report survey was administered via the Qualtrics platform during student attendance at the school. Qualtrics facilitates audio computer–assisted self-interviews for those who require this support. Organized by the project manager, data collection leaders, and SCAs, each school administers the survey at a predetermined date and time either inside computer laboratories or on student laptops while at school, depending on the best fitting circumstances at any given time. To date (wave 3), this process has sometimes required multiple days of data collection to acquire responses from the maximum number of participants. During the COVID-19 pandemic, added flexibility became an important feature of the study data collection procedures, as some students were only accessible while at home because of school closures or mixed or distance learning protocols. In such instances, survey data were collected from students while they were home via laptops during web-based class times and supervised by the SCA. Teacher surveys were administered using a paper-and-pencil questionnaire within schools supervised by the data collection leaders and SCAs. Informed consent was collected. On the basis of school preferences, ≥1 teacher would respond to the survey questions pertaining to each individual student linked to the database via students’ school ID numbers. A project manager who is not part of the investigative team entered the data into a spreadsheet deidentified by the honest broker before cleaning and analyses. The schools and BoEs submitted zip code and grade data to the honest broker.

### Compensation and Incentive Structure

To maximize buy-in and commitment from schools, study participants, and data collection personnel, an incentive system was built into all the data collection procedures. Incentives were provided after each wave of data collection, and the data collection leaders were hired part-time to oversee data collection within the schools. Schools are directly incentivized with US $1500 for their time and efforts, and the SCAs are compensated separately with US $500 to oversee the survey administration for their school. Teachers who respond to the teacher survey are compensated with US $10 for each student assessment they complete, and students are given a healthful treat (eg, Kind bar) and a pencil, pen, or other “swag” that includes the study logo as a token of appreciation for participating. Students are also enrolled in a lottery for an iPad, which is drawn at the end of each wave of data collection. During challenging times such as the COVID-19 period, this incentive system has proven vital for encouraging high levels of participation.

### Data Processing, Analysis Plan, Power Analysis

#### Overview

The data obtained from the students, teachers, and archives were organized and cleaned following well-established quality control procedures. Item distributions and construct psychometric properties are examined, and a comprehensive codebook is created, which is updated once each new wave of data becomes available. For multivariable tests, most analyses will be run in Mplus using maximum likelihood (ML) robust estimation, a sandwich estimator that generates estimation with SEs that are robust for nonnormal data, including symmetric or platykurtic, nonsymmetric, or zero kurtotic distributions. Mplus facilitates path analysis, latent variable modeling, and estimation of latent growth curves (LGC) and growth mixture models. Mplus can provide ML estimates for a range of distributions pertinent to alcohol use and psychosocial outcomes, including binary (lifetime onset of alcohol use), zero-inflated Poisson, negative binomial (low base rate count data; eg, days using alcohol), and continuous data. Model fit will be evaluated using the criteria for chi-square, comparative fit index, and root mean square error of approximation proposed by Hu and Bentler [69]. The study team has used all of these techniques in previous research [70-74]. Our analyses routinely incorporate nested data (youth nested in schools or youth nested in zip code). When data are clustered, Mplus adjusts the SEs via the multilevel pseudo ML estimator [75]. The method is likelihood based and thus applies to multivariate outcomes from any parametric family of distributions, including generalized linear models. The default estimation of Mplus is robust to bias, with a ratio of parameters to sample size of 1:5.15. In the event that the cell sizes are smaller than expected and the software encounters convergence problems or biased estimates, we will incorporate a Bayesian methodology, which does not assume large sample sizes and, in fact, performs well with very small sample sizes, particularly with informative priors [76,77].

In addition, Mplus provides unbiased ML parameter estimates and reasonable estimates of SEs for cases in which data are missing completely at random or missing at random [78,79]. Modeling data that are missing not at random is possible with full information ML estimation using latent indicators of missingness in a mixture model context, as specified by Muthén et al [80].

#### Power Analysis

Power was determined using Monte Carlo simulations in Mplus (version 7.4195), with a proposal based on our conceptual model (Figure 1) as an example. The proposal is a parallel LGC model with structural relationships with a time-invariant risk factor index and was estimated with an expected 2% to 4% attrition and ML under the assumptions of normality (although in the event of nonnormality, the proposed analytic adaptions will be made). Parameters were estimated as 0.5 for the intercept growth factors, 0.25 for the residual variance of the intercept growth factors, and 0.09 for the residual variance of the slope growth factor [81]. To determine the smallest, well-powered effects (0.80), regression coefficient values were estimated starting at 0.2 (reflecting a medium effect size) and decreasing for each subsequent test by 0.01. Tests of power for N=2200 were conducted for each model, with 1000 Monte Carlo simulations for each model. All 1000 simulations were completed successfully for each model. The following criteria were used to determine the smallest detectible effect: (1) parameters and SE biases ≤10% per parameter, (2) coverage ≥0.91, and (3) power for all main effect regression coefficient parameters had to be approximately 0.80 for sufficient power. A final model of regression coefficients equaling 0.07 was obtained with satisfactory model omnibus statistics: *χ*^2^_74_=74.5 (SE 11.92); root mean square error of approximation 0.003 (0.004); standardized root mean squared residual 0.013 (0.001). The 95% CI coverage for all parameters, as well as all the power estimates, was ≥0.942. We repeated this procedure for a discrete time survival mediation model using a conservative estimate of alcohol use onset from our prior research (8% in sixth grade, growing to 20% in eighth grade), a time-invariant risk index, and an LGC as mediators. All power estimates for model coefficients as small as 0.07 were ≥0.80, with satisfactory model omnibus statistics: *χ*^2^_55_=78.6 (25.259). The 95% CI coverage for all the parameters was ≥0.91.

### Ethics Approval

All study documents, plans, and protocols were reviewed and approved by WV University IRB. This included plans for the administration of the parent or caregiver letters, student surveys, teachers’ consent and surveys, incentive systems, individual-level confidentiality, data cleaning and data linking procedures, and analyses. The IRB of West Virginia University approved all the study protocols (#1903499093).

### Data-Sharing Plan

The proposed research does not exceed the US $500,000 cap set by the National Institutes of Health in any project year. However, based on the importance of the data, we encourage collaborations with interested investigators. We will also make deidentified data available to appropriate external investigators within 2 years of the publication of the main findings of the study. This will occur under the auspices of a data-sharing agreement that provides for the following: (1) a commitment to use the data only for research purposes and not to identify any individual participant, (2) a commitment to secure the data using appropriate computer technology, (3) a commitment to destroy or return the data after analyses are completed, and (4) monitoring by an approved human subjects board.

## Results

In 2020 and 2021, 3 waves of student data, 2 waves of teacher survey data, and zip codes for student homes via school records were collected. Table 2 shows a breakdown of the number of student surveys and adjusted response rates based on accessibility because of the COVID-19 pandemic.

The response rate of each wave exceeded 80%. Variations in sampling frame numbers are to be expected as the sampling frame encompasses all registered students in the 20 schools that participate in the study each year. Minimal changes from one semester to the next are considered normal. The inaccessible students were those who participated in the WV state curriculum during the COVID-19 pandemic and, thus, were not registered through any of the participating schools. Opt-outs are the eligible participants whose parents or caregivers or the students themselves opted to not participate in the study. Response rates are calculated based on the total number of cleaned responses (omitting double entries and empty entries) divided by the total number of eligible participants (total sampling frame minus inaccessible students). Opt-outs are included in the sampling frame when response rates are calculated.

**Table 2:** Breakdown of participant numbers for all study waves 2020 to 2021.

Wave	Sampling frame, N	Inaccessible (COVID-19), n (%)	Opt-outs, n (%)	Cleaned responses, n (%; percentage of sampling frame, including inaccessible)	Response rate (%; percentage of sampling frame minus inaccessible)
1 (fall 2020)	2247	576 (25.63)	79 (3.52)	1349 (60.04)	80.7
2 (spring 2021)	2320	425 (18.32)	83 (3.58)	1649 (71.08)	87
3 (fall 2021)	2374	78 (3.29)	73 (3.07)	1909 (80.41)	83.1

## Discussion

### Study Overview

The YMHS plans to fill a gap in the literature concerning the health and well-being of early adolescents residing in geographically isolated and often resource-scarce Appalachian communities. The proposed research focuses on alcohol use among youth in small cities, towns, and rural areas of WV, a US state entirely within the Appalachian region. Historically marginalized in American society, many Appalachian communities experience chronic poverty and epidemic levels of opioid abuse [5]. In the past, residing in rural communities protected youth from many risk factors of early onset alcohol use encountered in urban settings. However, recent research underscores the prevalence of alcohol use in adolescents and alcohol use disorders in adults in resource-poor rural communities [9]. Of particular concern, early onset alcohol use places rural youth at risk for future substance use, including opioid abuse [13-15]. We will follow a single cohort of middle school students in 5 counties and 20 schools in WV as they progress through the middle school years from sixth to eighth grade. A total of 6 waves of data will be collected in the fall and spring each year via self-reported surveys, teacher surveys, and archival school records. To date, 3 waves of data have been collected, with a response rate of ≥80% for each wave. Data cleaning, quality checks, and preliminary analyses have been conducted.

We believe that our network of data collection personnel, relationship with county and school officials, and incentive structure have all contributed to the consistently high response rate we have achieved, despite the major challenges brought about by the COVID-19 pandemic. Through contracted agreements with county-level BoEs and superintendents, locally hired data collection leaders, and principals and SCAs in all schools, we were able to adjust and readjust our plans for data collection with many schools during the height of the COVID-19 pandemic (specifically, in the fall of 2020 and spring of 2021) and secure solid responses from most students. Currently, we have 3 manuscripts under review from the first 3 waves of the data, others in preparation or planning, and multiple ideas at the analysis stage, including those that directly test the conceptual model in the grant application.

### Limitations and Strengths

During the COVID-19 pandemic, schools have been stretched thin to continue providing federal and state-mandated services to children. Despite being able to navigate many challenges that resulted from the pandemic, a noteworthy complication in our data collection efforts to date concerns issues caused by a higher than expected number of missing participants, particularly from wave 1 (at the height of the pandemic in the fall of 2020 and before vaccines became widely available) and wave 2 (in the spring of 2021 when vaccines were only recently available and not yet widely distributed). Although our response rate was >80% for waves 1 and 2, we operated with a somewhat condensed sampling frame in both instances (Table 2). During the pandemic in WV, as in most other US states, students were given three options to register for their studies: in school only, hybrid format (enrolling in school but studying from home), and via the state-operated curriculum not executed via individual schools. In addition, homeschooling remained an option. Although our sampling frame is contingent on school collaborations, and thus, the sampling frame for each wave represents all accessible students in each of the 20 participating schools, unfortunately, we missed students who participated in the WV state-operated curriculum. A challenging and persistent problem in any longitudinal study is how to deal with dropout rates that may be higher than expected via random missingness. Owing to the COVID-19 pandemic, moving forward, an important problem for the study team will be dealing with missingness that is not because of systematic error. In particular, given that participants who were included in the original sampling frame may have elected to participate in the state-operated curriculum, they may not have been randomly distributed in the sample.

Despite notable shortcomings that were predominantly brought about by the COVID-19 pandemic, our study design and data collection system have several strengths. So far, we have been able to secure 3 waves of high-quality data (complete responses) from approximately 1300 to 1900 middle school–aged youth. Our approach to data collection mimics the principles of community engagement, in which collaborations with locally knowledgeable and trusted personnel have been established to secure access to participants and their caregivers. A considerable portion of the grant funding is used to incentivize both participants and school and county personnel who assist us in our data collection efforts. In addition, each county and school receives a summary report with aggregated results regarding risk and protective factors and alcohol, tobacco, and other drug use outcomes among their youth, which they can then use to plan prevention efforts or apply for funding. This system has proved vitally important during the COVID-19 pandemic when many competing interests have been brought to the table of county and school administrators to keep our education systems functioning. The honest broker system has proven to be an important and novel approach to securing linkable longitudinal data from many participants without jeopardizing confidentiality.

### Conclusions

The YMHS cohort will provide novel data for both risk and protective behaviors and social contexts among early adolescents residing in the Appalachian region of the United States. The data collection mechanisms in this study comprise a combination of self-reported student surveys, teacher surveys, and archival school data. This study is unique in that it follows a young group of adolescents through an understudied developmental period in an environment that has not received ample attention to date. Through a robust data collection structure and collaborations with county and school officials, our team has collected 3 waves of data with high response rates despite numerous challenges brought on by the COVID-19 pandemic. Data from this study will provide unique opportunities for various analyses related to early adolescent health and development, with a focus on the Appalachian environment.

## Appendix

Multimedia Appendix 1 Peer-review report by the Community Influences on Health Behavior Study Section - Healthcare Delivery and Methodologies Integrated Review Group (National Institutes of Health, USA).

## Abbreviations

BoE: Board of Education
IRB: institutional review board
LGC: latent growth curve
ML: maximum likelihood
MoU: Memorandum of Understanding
SCA: Supervising Contact Agent
WV: West Virginia
YMHS: Young Mountaineer Health Study
