# Supplementary material for: Risk and Resilience Pathways, Community Adversity, Decision-making, and Alcohol Use Among Appalachian Adolescents: Protocol for the Longitudinal Young Mountaineer Health Study Cohort
Source: JMIR Res Protoc. 2022 Aug 5;11(8):e40451. doi: 10.2196/40451 (PMC9391973; doi:10.2196/40451)
Supplement: Multimedia Appendix 1 [file resprot_v11i8e40451_app1.pdf]

SUMMARY STATEMENT

(Privileged Communication)

PROGRAM CONTACT:
Beverly Ruffin
301-443-0281
beverly.ruffin@nih.gov

Release Date: **02/21/2019**
Revised Date:

Application Number: **1 R01 AA027241-01A1**

Principal Investigator

KRISTJANSSON, ALFGEIR

Applicant Organization: WEST VIRGINIA UNIVERSITY

Review Group: **CIHB**
Community Influences on Health Behavior Study Section

Meeting Date: **02/07/2019**
Council: **MAY 2019**
Requested Start: **07/01/2019**
RFA/PA: **PA18-390**
PCC: **AE VD**

Project Title: **Risk and resilience pathways linking community adversity, decision making, and alcohol misuse: A prospective study of Appalachian adolescents**
SRG Action: **Impact Score:20 Percentile:5**
Next Steps: **Visit https://grants.nih.gov/grants/next_steps.htm**
Human Subjects: 48-At time of award, restrictions will apply
Animal Subjects: 10-No live vertebrate animals involved for competing appl.
Gender: 1A-Both genders, scientifically acceptable
Minority: 1A-Minorities and non-minorities, scientifically acceptable
Children: 1A-Both Children and Adults, scientifically acceptable

Project Year	Direct Costs Requested	Estimated Total Cost
1	384,382	570,489
2	437,404	649,183
3	461,821	685,422
4	369,300	548,105
5	297,836	442,040
TOTAL	1,950,743	2,895,240

ADMINISTRATIVE BUDGET NOTE: The budget shown is the requested budget and has not been adjusted to reflect any recommendations made by reviewers. If an award is planned, the costs will be calculated by Institute grants management staff based on the recommendations outlined below in the COMMITTEE BUDGET RECOMMENDATIONS section.
EARLY STAGE INVESTIGATOR
NEW INVESTIGATOR

1R01AA027241-01A1 Kristjansson, Alfgeir

EARLY STAGE INVESTIGATOR
NEW INVESTIGATOR
PROTECTION OF HUMAN SUBJECTS UNACCEPTABLE

RESUME AND SUMMARY OF DISCUSSION: This application proposes to longitudinally examine the role of environmental, social, and personal factors affecting alcohol use among middle school students in the Appalachia area of West Virginia. The panel found the study highly significant and innovative in focusing on the integration of contextual risk and protective factors with psychological and interpersonal factors, and on assessing early alcohol use which can affect long term alcohol use. Additionally, the inclusion of caffeine use, sleep problems, and family school and community level protective factors is novel. The study is using an innovative process with local schools that allows for the creation of deidentified data at the student and zip code level. This process could provide a model for other school-based research. The team is highly qualified and has well established relationships with the schools. Additional strengths include well-articulated measures, a detailed analysis plan, and robust power calculations. The resubmission is responsive to the prior reviews and documents the variability between schools and accounts for potential differences between teacher and student perceptions. Reviewers noted minor weaknesses including little attention to the triangulation of data, the lack of preliminary data to back up expected retention rates, and inadequate measurement of alcohol use among other family members. Overall, the panel found that a study examining factors affecting alcohol use among middle school students is very significant and this project will have a high impact.

DESCRIPTION (provided by applicant): Early adolescent alcohol use impairs psychosocial and neurocognitive development and increases vulnerabilities to drug abuse, academic failure, and mental health problems. The proposed research focuses on youth alcohol use in rural areas of West Virginia (WV), within the Appalachian region. Marginalized in American society, many Appalachian communities suffer chronic poverty and epidemic levels of opioid abuse. Emerging neurocognitive research stresses the maturation and integration of reward-salience and cognitive control systems associated with adolescent emotion regulation and healthy decision-making abilities. Development in decision making systems forecasts alcohol misuse, however, the factors that contribute to differences in decision making trajectories are poorly understood. Also, accumulating research by our team and others links widespread use of caffeinated beverages by rural youth to alcohol use vulnerabilities. A psychostimulant possessing arousal, motor activation, and reinforcing proper- ties, even moderate doses of caffeine produce symptoms in children and adolescents, including headache, nausea, drowsiness, fatigue, mood disturbances, and sleeplessness. Despite these dangers, prospective investigations of routine caffeine use and the mechanisms through which it confers alcohol use vulnerability are virtually non-existent. We propose to investigate contextual protective factors hypothesized to attenuate the influence of community disadvantage on negative emotionality, maladaptive health behavior, and decision-making trajectories. These factors include regulated, affectively positive family environments, health promoting classroom and school environments, and collective efficacy in local communities. We propose to address these issues in a prospective study of ~2,400 middle school youth from a range of rural, small town, and small city (< 30k) public schools in WV. Our specific aims are: 1. To test pathways linking community disadvantage to alcohol misuse. We expect community disadvantage to forecast slower growth in decision making processes directly and indirectly via negative emotionality which is reinforced by use of caffeine and attendant problems with sleep. In turn, we expect negative emotionality and decision-making trajectories to forecast alcohol misuse via proximal vulnerability factors (affiliation with risky peers, academic disengagement). 2. To test hypotheses of contextual factors that protect youth from community disadvantage. We expect contextual protective factors to attenuate the influence of community disadvantage on (a) negative

emotionality, (b) caffeine use, sleep problems, and (c) decision making. 3. To explore these alcohol antecedents in the onset of nicotine, marijuana, and other drug use.

PUBLIC HEALTH RELEVANCE: This study focuses on environmental, social, and personal reasons for alcohol use onset in a group of middle school students that reside in rural areas of Appalachia. Specifically, the study aims to assess the relations between community risk factors, such as rural poverty and norms supportive of youth alcohol use, with emotional well-being, caffeine use, decision making processes, and alcohol use onset and progression in young adolescents.

CRITIQUE 1

Significance: 3
Investigator(s): 1
Innovation: 1
Approach: 3
Environment: 1

Overall Impact: The authors propose a longitudinal study to investigate antecedents of alcohol use among middle school students in Appalachia, drawing from a sample of 2400 students from a wide range of public schools in West Virginia. Six waves of data collection over a two-year period would be analyzed to provide a more nuanced understanding specific mechanisms that underlie the link between community disadvantage and alcohol use among adolescents. Based on a well-developed conceptual model, the authors hypothesize that community disadvantage contributes to slow growth and maturation in decision-making processes, and that this relationship is also mediated through negative emotionality, caffeine use, and sleep problems. These combined factors impact proximal alcohol use mechanisms (academic engagement, risky peer affiliations and norms) as well as alcohol (and other substance) use. The integration of both ecological and neurocognitive perspectives is innovative, and the inclusion of caffeine use and sleep problems as risk factors is particularly novel. Investigation of these risk factors, in addition to family, school and community level protective factors proposed in the study, are likely to yield findings that may have moderate to high impact on prevention and early intervention strategies for youth in low-income and rural communities. Furthermore, the PI/team have developed an innovative process with local schools, which allows for creation of de-identified data that links self-report data with school records and census zipcodes (called the “honest broker” system). This approach may provide a useful model for future school-based research. The likelihood of successful implementation is strong given the highly qualified team, solid preliminary research, and well-established collaborative relationships with schools in the region. A few weaknesses in approach remain in the resubmission, including unknown risk of attrition, racial/ethnic homogeneity of the sample (88% White), and absence of measures related to school-level student engagement strategies. Despite weaknesses, the overall potential to advance understanding of how these multiple factors influence the relationship between community disadvantage and alcohol use is high.

1. Significance:

Strengths

- Disparities in risk of alcohol use and alcohol use disorders among youth in low-income rural communities and small towns remains an important public health issue.
- Early onset alcohol use place rural youth at risk for later alcohol and drug related problems, including risk for opioid use.

- The longitudinal design, and the integration of contextual risk and protective factors with psychological and interpersonal factors, is likely to yield results that may be used to refine prevention strategies in schools and communities.
- Examining the role caffeine use and sleep deprivation in alcohol misuse is particularly unique and may pave the way for new intervention strategies, particularly given preliminary research by the authors suggesting that these factors may predict alcohol use and issues with academic engagement.
- Rationale and supportive evidence for diverse measures are well-developed.

Weaknesses

- Minor. Contextual protective factors (i.e. protective parenting, neighborhood collective efficacy and safety), are assessed primarily through youth self-report and through the perspective of teachers (e.g., school and classroom climate). This may still introduce bias, although the authors provide some reassuring data about the psychometric properties and predictive validity of these measures in the resubmission.

2. Investigator(s):

Strengths

- The PI is well qualified, has a strong track-record in publication on adolescent substance use, and has already institutionalized collaborative relationships with local schools in the region.
- The other members of the research team possess complementary skills, are each well-qualified, and have a strong history of collaboration with the PI.
- Although some co-Investigators reside well outside of the area, they describe a viable plan for communicating regularly via technology (e.g. Skype).

Weaknesses

- None noted.

3. Innovation:

Strengths

- The concurrent investigation of contextual risk and protective factors with other psychological and interpersonal factors (decision making processes, negative emotionality, academic engagement/achievement, and peer affiliations community level indicators of disadvantage and disorganization) is a significant innovation.
- The inclusion of caffeine uses and suboptimal sleep as behavioral mechanisms that influence risk for alcohol use is novel and highly likely to provide useful information given regional caffeine use norms and opportunities and high levels of caffeinated drink consumption.
- The “honest broker” system that the PI/team have developed with local schools is innovative and may provide a model for other research. (The system allows for creating de-identified data while linking self-report and school record data including zip code information).

Weaknesses

- The use of the central Appalachian sample, although useful for the study, is not particularly innovative.

4. Approach:

Strengths

- The conceptual model provides a coherent and research-based rationale for the study hypotheses.
- Prospective longitudinal study design.
- The use of an "honest broker system" that facilitates creation of a de-identified dataset that includes self-report data, school record data, and census-linked zip code data.
- The current study is unique but builds on a solid foundation of prior research. For example, solid preliminary research provides a strong rationale for the variables and hypotheses of the study (e.g., establishing pathways between caffeine consumption, inadequate sleep, school engagement and alcohol use frequency).
- Well-established partnerships with schools have resulted in strong recruitment rates and quality of data in past studies.
- The analysis plan is well-described and sound.
- Power analysis provided evidence for adequacy of the sample size.
- Several key methodological concerns are addressed in the resubmission, including providing evidence for the variability between schools, alternative analysis plans if cell sizes are smaller than expected or if teacher and youth reports diverge, and clarity about how the team will test resilience hypotheses.
- The team will explore whether sex moderates the pathways in the conceptual model.

Weaknesses

- Minor. Contextual protective factors (i.e. protective parenting, neighborhood collective efficacy and safety), are assessed primarily through youth self-report and through the perspective of teachers (e.g., school and classroom climate). This may still introduce bias, although the authors provide some reassuring data about the psychometric properties and predictive validity of these measures in the resubmission.
- Minor. Although the retention plan is well-described and strong relationships with schools are an asset, unexpected attrition could undermine the impact of the study.
- Minor. Some concerns raised by reviewers of the original proposal are not fully addressed, such as selection into neighborhoods and characteristics/activities of schools that might foster student engagement.
- Minor. The sample appears to be fairly homogenous in terms of race/ethnicity (88% White). Although this is demographically similar to the region, the capacity to explore possible differences by race/ethnicity is low.
- Although the longitudinal design is a strength, it may be difficult to detect changes cognitive and emotional changes in the two-year time frame.

5. Environment:

Strengths

- The study will be implemented through the Rural Health and Youth Development Center of West Virginia University (WVU), an interdisciplinary research center directed by the PI. The center has (since its inception in 2012) involved more than 30,000 adolescents in research on the health and well-being of Appalachian youth.
- WVU (R1, land-grant university) and the WVU School of Public Health provide a sound intellectual environment and offer sufficient resources to support the grant.

- The team has well-developed relationships with middle school districts and local communities throughout the region, evidenced by letters of support from appropriate representatives.

Weaknesses

- None noted.

Protections for Human Subjects:

Acceptable Risks and/or Adequate Protections

- Appropriate protections for research participants are described

Data and Safety Monitoring Plan (Applicable for Clinical Trials Only):

Inclusion of Women, Minorities and Children:

- Sex/Gender: Distribution justified scientifically
- Race/Ethnicity: Distribution justified scientifically
- For NIH-Defined Phase III trials, Plans for valid design and analysis:
- Inclusion/Exclusion of Children under 18: Including ages <18; justified scientifically
- Middle school age children are the primary focus of the study.

Vertebrate Animals:

Not Applicable (No Vertebrate Animals)

Biohazards:

Not Applicable (No Biohazards)

Resubmission:

- The authors re-wrote the proposal as were thorough in addressing the concerns and recommendations of reviewers. The authors address concerns about limitations of youth self-reports by including data on perspectives from teachers, explaining biases inherent in parent perspectives, and describing several revisions in analyses plans that strengthen use of youth measures. They also provide evidence for community and school variability and explain how the findings may be generalizable to other rural, low-resources areas. A few concerns remain, but the rigor of the project appears to have been substantially improved (and clarified) in the revision. The potential impact in the resubmission is high.

Applications from Foreign Organizations:

Not Applicable (No Foreign Organizations)

Select Agents:

Not Applicable (No Select Agents)

Resource Sharing Plans:

Acceptable

Authentication of Key Biological and/or Chemical Resources:
Not Applicable (No Relevant Resources)

Budget and Period of Support:
Recommend as Requested

CRITIQUE 2

Significance: 2
Investigator(s): 1
Innovation: 2
Approach: 2
Environment: 1

Overall Impact: This is a longitudinal prospective study that seeks to determine the factors predict alcohol use such as negative emotionality, caffeine use and sleep problems, and decision-making trajectories in a low-income rural population. This proposal is significant as it addresses the early-onset of these issues in middle school population where symptoms first appear and are ripe for prevention and intervention. Additional strengths include combination of objective neurocognitive measures as well as sleep and caffeine intake (a significant issue in their particular population). A minor weakness includes additional reporting of alcohol use beyond self-report, though authors make a strong case for not including parent report. Inclusion of more alcohol and/or substance use measures may strengthen the application in the absence of additional reporters.

1. Significance:

Strengths

- This a longitudinal prospective study addresses a health disparity in a low-income often underrepresented population.
- Addressing factors that predict early-onset alcohol in low income and isolated populations is necessary to address adolescent and adult substance use.
- Preliminary studies provide strong evidence for this work.

Weaknesses

- The generalizability of the work may be limited to Appalachia, though the application makes the case for any resource poor population.

2. Investigator(s):

Strengths

- The PI is well published in adolescent substance use and the other foci of this paper, including caffeine and behavioral health. She also founded the RHYDC.

- Inclusion of Dr. Lily through the West Virginia Clinical Translational Science Institute (WVCTSI) Clinical Research Design, Epidemiology, and Biostatistics (CRDEB) Program who has previously worked with datasets from Appalachia.
- An excellent group of international investigators to support all aspects of the project.
- Support from Calhoun County schools is important.

Weaknesses

- None noted.

3. Innovation:

Strengths

- This application will simultaneously address neurocognition, community factors and substance use behaviors of middle school students.
- The inclusion of sleep and cognition in this model are unique.

Weaknesses

- Alcohol use reported by others would strengthen the application, though the authors rationale for not including parents is strong, another reporter on use would validate the self-report.

4. Approach:

Strengths

- The addition of teacher report to the student self-report is a strength and there is a plan for missing data.
- There waves of data collection a year will yield a rich dataset.
- Addresses a large sample size and considers both risk and resilience factors.
- Use of neurocognitive battery done by researchers is a strength.

Weaknesses

- More information is needed on the alcohol and substance use measures. And if alcohol use is the outcome then perhaps more than 1 measure is needed.
- Measures of sleep and caffeine intake are self-report.
- Stronger dissemination plan is needed given community-engaged nature of the study.
- Unclear if data for retention was based on preliminary data within this population specifically or just overall estimates.

5. Environment:

Strengths

- WVU has the research resources needed to carry out such a large project.
- The PI is the director for the West Virginia University's (WVU) Rural Health and Youth Development Center (RHYDC), an interdisciplinary center composed of public health, psychology, education, and sociology researchers who focus on the health and well-being of Central Appalachian youth.

Weaknesses

- None noted.

Protections for Human Subjects:

Acceptable Risks and/or Adequate Protections

- Acceptable

Data and Safety Monitoring Plan (Applicable for Clinical Trials Only):

Not Applicable (No Clinical Trials)

Inclusion of Women, Minorities and Children:

- Sex/Gender: Distribution justified scientifically
- Race/Ethnicity: Distribution justified scientifically
- For NIH-Defined Phase III trials, Plans for valid design and analysis: Not applicable
- Inclusion/Exclusion of Children under 18: Including ages <18; justified scientifically
- focus on adolescents is well justified

Vertebrate Animals:

Not Applicable (No Vertebrate Animals)

Biohazards:

Not Applicable (No Biohazards)

Resubmission:

- The application clearly addressed all reviewer comments well, including the addition of teacher report measures, how this work was different from and expanded past work, clarifying that the Appalachian region is an ideal context for testing alcohol and bolstering of the plan to measure protective factors in youth

Applications from Foreign Organizations:

Justified

- Included Professor from Iceland

Select Agents:

Not Applicable (No Select Agents)

Resource Sharing Plans:

Acceptable

- Acceptable

Authentication of Key Biological and/or Chemical Resources:

Not Applicable (No Relevant Resources)

Budget and Period of Support:

Recommend as Requested

- No concerns noted

CRITIQUE 3

Significance: 3
Investigator(s): 1
Innovation: 1
Approach: 3
Environment: 1

Overall Impact: The proposal which focuses on an innovative selection of mutable factors including caffeine, sleep hygiene, contextual factors influencing decision-making maturation are assessed longitudinally in association with alcohol and other substance use among Appalachian youth. The investigator team is very strong as is the environment which nicely align with the target population and proposed interdisciplinary methods. The premise and rigor are overall moderate to strong with minor weaknesses noted.

1. Significance:

Strengths

- Alcohol use among rural youth identified as relatively recent risk group with known differences with urban counterparts though understudied in this context.
- Need for broader understanding of how alcohol risk relates to factors such as caffeine overconsumption (which is prevalent in these rural communities among youth) and related sleep disturbance which might broaden the targets of interventions.
- Well-developed conceptual model which supports scientific premise and nicely captures the relationship among contextual risk/protective factors, decision-making, caffeine and sleep, and other known alcohol use risk factors (e.g., peer use, norms, etc.). Each of these domains are well supported in the significance section.

Weaknesses

- Somewhat muted significance due to lack of parental data collection to triangulate parental contextual measures and perhaps other contextual measures. It is understood why investigators opted not to incorporate this based on the drawbacks discussed.

2. Investigator(s):

Strengths

- Investigative team has a good track record of collaboration and work with Appalachian youth with respect to substance use and psychosocial adjustment. Interdisciplinary expertise which dovetail with proposed study focus and methods.
- Complementary group of external investigators who buttress the expertise of the local investigators in areas of caffeine effects and relationship with alcohol use; longitudinal modeling

of community and family contexts on youth behavior regulation; adolescent and young adult decision-making processes as it relates to alcohol use.

- Plan to connect biweekly or more frequently for project management.

Weaknesses

- None noted.

3. Innovation:

Strengths

- Well-articulated novel aspects outlined in the innovation section including study population of adolescents in WV, encompassed in Central Appalachia; integrating the maturation of decision-making capacity with risk/protective factors in multiple contexts; caffeine +/- sleep disruption with alcohol use risk; and 'honest broker' system of school-based data collection regionally based on 8-year collaboration and trust-building partnership between schools and researchers.

Weaknesses

- None noted.

4. Approach:

Strengths

- Given prior research with these youth at these schools, good data presented on anticipated sample size and retention over the proposed 6 waves (83.3%) which is admirable.
- Self-reported data quality is improved by using ACASI, spacing collection over 2 days, and good buy-in from schools/teachers with high participation rate among the student body.
- Complementary data from school records (grades), teacher report, and contextual data from Census using residential address.
- Sex as a biological variable addressed.

Weaknesses

- Not clear whether aggregated youth responses might be used as a contextual or school-level variable. If not, this might be of value in relaying norms in attitudes and behaviors.
- Unclear how all of the outcome measures (various measures relating to self-reported alcohol and other substances as well as school infractions relating to substance use) will be used together or separately in the various models.

5. Environment:

Strengths

- Very strong ties with WV secondary schools with good track record of data collection in collaboration with the schools an 'honest broker'. Letters of support from all schools.
- WVU Rural Health and Youth Development Center, led by Kistjansson and in existence since 2012, is an interdisciplinary group of researchers with support of this research focused on Appalachian youth health issues.

Weaknesses

- None noted.

Study Timeline:

Strengths

- Present and appropriate.

Weaknesses

- None noted.

Protections for Human Subjects:

Unacceptable Risks and/or Inadequate Protections

- Minor note: Another risk might be loss of confidentiality due to statistical disclosure control, particularly given the predominant Caucasian community and perhaps sparsely populated areas (1 black youth in a particular census tract thus identifying him/her).

Data and Safety Monitoring Plan (Applicable for Clinical Trials Only):

Not Applicable (No Clinical Trials)

Inclusion of Women, Minorities and Children:

- Sex/Gender: Distribution justified scientifically
- Race/Ethnicity: Distribution justified scientifically
- For NIH-Defined Phase III trials, Plans for valid design and analysis: Not applicable
- Inclusion/Exclusion of Children under 18: Including ages <18; justified scientifically

Vertebrate Animals:

Not Applicable (No Vertebrate Animals)

Biohazards:

Not Applicable (No Biohazards)

Resubmission:

- Nicely addressed reviewer's comments with edits to the proposal and/or explanations in the intro section.

Applications from Foreign Organizations:

Not Applicable (No Foreign Organizations)

Select Agents:

Not Applicable (No Select Agents)

Resource Sharing Plans:

Not Applicable (No Relevant Resources)

Authentication of Key Biological and/or Chemical Resources:

Not Applicable (No Relevant Resources)

Budget and Period of Support:

Recommend as Requested

THE FOLLOWING SECTIONS WERE PREPARED BY THE SCIENTIFIC REVIEW OFFICER TO SUMMARIZE THE OUTCOME OF DISCUSSIONS OF THE REVIEW COMMITTEE, OR REVIEWERS' WRITTEN CRITIQUES, ON THE FOLLOWING ISSUES:

PROTECTION OF HUMAN SUBJECTS: UNACCEPTABLE
Reviewers noted that the potential loss of confidentiality due to statistical disclosure control, and appropriate protections for such risks are not fully addressed.

INCLUSION OF WOMEN PLAN: ACCEPTABLE

INCLUSION OF MINORITIES PLAN: ACCEPTABLE

INCLUSION OF CHILDREN PLAN: ACCEPTABLE

COMMITTEE BUDGET RECOMMENDATIONS: The budget was recommended as requested.

Footnotes for 1 R01 AA027241-01A1; PI Name: Kristjansson, Alfgeir

NIH has modified its policy regarding the receipt of resubmissions (amended applications). See Guide Notice NOT-OD-14-074 at http://grants.nih.gov/grants/guide/notice-files/NOT-OD-14-074.html. The impact/priority score is calculated after discussion of an application by averaging the overall scores (1-9) given by all voting reviewers on the committee and multiplying by 10. The criterion scores are submitted prior to the meeting by the individual reviewers assigned to an application, and are not discussed specifically at the review meeting or calculated into the overall impact score. Some applications also receive a percentile ranking. For details on the review process, see http://grants.nih.gov/grants/peer_review_process.htm#scoring.

MEETING ROSTER

Community Influences on Health Behavior Study Section
Healthcare Delivery and Methodologies Integrated Review Group
CENTER FOR SCIENTIFIC REVIEW
CIHB
02/07/2019 - 02/08/2019

Notice of NIH Policy to All Applicants: Meeting rosters are provided for information purposes only. Applicant investigators and institutional officials must not communicate directly with study section members about an application before or after the review. Failure to observe this policy will create a serious breach of integrity in the peer review process, and may lead to actions outlined in NOT-OD-14-073 at https://grants.nih.gov/grants/guide/notice-files/NOT-OD-14-073.html and NOT-OD-15-106 at https://grants.nih.gov/grants/guide/notice-files/NOT-OD-15-106.html, including removal of the application from immediate review.

CHAIRPERSON(S)

HORN, KIMBERLY A, EDD
ASSOCIATE VICE PRESIDENT OF CLINICAL RESEARCH COLLABORATIVES
OFFICE OF THE VICE PRESIDENT OF HEALTH SCIENCES AND TECHNOLOGY
VIRIGINA POLYTECHNIC INSTITUTE AND STATE UNIVERSITY
BLACKSBURG, VA 24061

MEMBERS

ALLEN, MICHELE L, MD *
ASSOCIATE PROFESSOR
DEPARTMENT OF FAMILY MEDICINE AND COMMUNITY HEALTH
PROGRAM IN HEALTH DISPARTIES RESEARCH
UNIVERSITY OF MINNESOTA
MINNEAPOLIS, MN 55414

BOOTH, ROBERT EDWIN, PHD *
PROFESSOR
DEPARTMENT OF PSYCHIATRY
SCHOOL OF MEDICINE
UNIVERSITY OF COLORADO
DENVER, CO 80218

BRUGGE, DOUG, PHD
PROFESSOR
DEPARTMENT OF PUBLIC HEALTH AND COMMUNITY MEDICINE
TUFTS UNIVERSITY SCHOOL OF MEDICINE
BOSTON, MA 02111

BRUNST, KELLY J, PHD *
ASSISTANT PROFESSOR
UNIVERSITY OF CINCINNATI
CINCINNATI, OH 45267

CAVAZOS-REHG, PATRICIA A, PHD
ASSOCIATE PROFESSOR
DEPARTMENT OF PSYCHIATRY
WASHINGTON UNIVERSITY SCHOOL OF MEDICINE
ST. LOUIS, MO 63110

COOPER, HANNAH L, SCD
PROFESSOR
DEPARTMENT OF BEHAVIORAL SCIENCES AND HEALTH EDUCATION
ROLLINS SCHOOL OF PUBLIC HEALTH
EMORY UNIVERSITY
ATLANTA, GA 30322

CORLISS, HEATHER L, PHD
PROFESSOR
DIVISION OF HEALTH PROMOTION AND BEHAVIORAL SCIENCE
SCHOOL OF PUBLIC HEALTH
SAN DIEGO STATE UNIVERSITY
SAN DIEGO, CA 92123

D'ANNA-HERNANDEZ, KIMBERLY LYNN, PHD *
ASSISTANT PROFESSOR
DEPARTMENT OF PSYCHOLOGY
CALIFORNIA STATE UNIVERSITY
SAN MARCOS, CA 92027

DRABBLE, LAURIE ANNE, PHD
PROFESSOR
SCHOOL OF SOCIAL WORK
SAN JOSE STATE UNIVERSITY
SAN JOSE, CA 95192

DUBOWITZ, TAMARA, SCD
SENIOR POLICY RESEARCHER
FACULTY PARDEE RAND GRADUATE SCHOOL
RAND CORPORATION
PITTSBURGH, PA 15213

DUNCAN, GLEN E, PHD *
PROFESSOR AND CHAIR
DEPARTMENT OF NUTRITION AND EXERCISE PHYSIOLOGY
ELSON S. FLOYD COLLEGE OF MEDICINE
UNIVERSITY OF WASHINGTON
SEATTLE, WA 98195

GOEBERT, DEBORAH A, DPH *
PROFESSOR
DEPARTMENT OF PSYCHIATRY
JOHN A. BURNS SCHOOL OF MEDICINE
UNIVERSITY OF HAWAII
HONOLULU, HI 96813

HIRATSUKA, VANESSA Y, PHD *
SENIOR RESEARCHER
SOUTHCENTRAL FOUNDATION
ANCHORAGE, AK 99508

KEYHANI, SALOMEH, MD *
PROFESSOR-IN-RESIDENCE
DIVISION OF GENERAL INTERNAL MEDICINE
UNIVERSITY OF CALIFORNIA AT SAN FRANCISCO
SAN FRANCISCO, CA 94121

KILE, MOLLY L, SCD *
ASSOCIATE PROFESSOR
COLLEGE OF PUBLIC HEALTH AND HUMAN SCIENCES
OREGON STATE UNIVERSITY
CORVALLIS, OR 97331

LI, WENJUN, PHD
PROFESSOR
DEPARTMENT OF MEDICINE
UNIVERSITY OF MASSACHUSETTS MEDICAL SCHOOL
WORCESTER, MA 01655

LING, PAMELA MAY, MD
PROFESSOR
DEPARTMENT OF MEDICINE
UNIVERSITY OF CALIFORNIA, SAN FRANCISCO
SAN FRANCISCO, CA 94143

MARTINEZ-DONATE, ANA P, PHD *
ASSOCIATE PROFESSOR
DEPARTMENT OF COMMUNITY HEALTH AND PREVENTION
DORNSIFE SCHOOL OF PUBLIC HEALTH
DREXEL UNIVERSITY
PHILADELPHIA, PA 19104

MARTINS, SILVIA SABOIA, MD, PHD
ASSOCIATE PROFESSOR
DEPARTMENT OF EPIDEMIOLOGY
MAILMAN SCHOOL OF PUBLIC HEALTH
COLUMBIA UNIVERSITY
NEW YORK, NY 10032

METRIK, JANE, PHD *
ASSOCIATE PROFESSOR
BEHAVIORAL AND SOCIAL SCIENCES
CENTER FOR ALCOHOL AND ADDICTION STUDIES
SCHOOL OF PUBLIC HEALTH
BROWN UNIVERSITY
PROVIDENCE, RI 02912

MOJTABAI, RAMIN, MD, PHD *
ASSOCIATE PROFESSOR
DEPARTMENT OF MENTAL HEALTH
JOHNS HOPKINS BLOOMBERG SCHOOL OF PUBLIC HEALTH
BALTIMORE, MD 21205

MOORE, ROLAND S, PHD
SENIOR RESEARCH SCIENTIST AND CENTER DIRECTOR
PREVENTION RESEARCH CENTER
PACIFIC INSTITUTE FOR RESEARCH AND EVALUATION
BERKELEY, CA 94704

PAN, WEI, PHD *
ASSOCIATE PROFESSOR
METHODS AND ANALYTICS RESEARCH AREA OF
EXCELLENCE
SCHOOL OF NURSING
DUKE UNIVERSITY
DURHAM, NC 27710

PINTO, ROGERIO M, PHD
ASSOCIATE PROFESSOR
SCHOOL OF SOCIAL WORK
UNIVERSITY OF MICHIGAN
ANN ARBOR, MI 48109

RAMASWAMY, MEGHA, PHD
ASSOCIATE PROFESSOR
DEPARTMENT OF PREVENTIVE MEDICINE
AND PUBLIC HEALTH
UNIVERSITY OF KANSAS SCHOOL OF MEDICINE
KANSAS CITY, KS 66160

SCHAIDER, LAUREL A., PHD *
RESEARCH SCIENTIST
ENVIRONMENTAL CHEMISTRY AND ENGINEERING
SILENT SPRING INSTITUTE
NEWTON, MA 02460

SCHOENBERG, NANCY E, PHD
MARION PEARSALL ENDOWED PROFESSOR
DEPARTMENT OF BEHAVIORAL SCIENCE
COLLEGE OF MEDICINE
UNIVERSITY OF KENTUCKY
LEXINGTON, KY 40536

STRONG, LARKIN, PHD *
ASSISTANT PROFESSOR
HEALTH DISPARITIES RESEARCH
UT MD ANDERSON CANCER CENTER
HOUSTON, TX 77230

VALDEZ, AVELARDO, PHD
PROFESSOR
DEPARTMENT OF SOCIAL WORK AND SOCIOLOGY
UNIVERSITY OF SOUTHERN CALIFORNIA
LOS ANGELES, CA 90089

WIEHE, SARAH ELIZABETH, MD, MPH
ASSOCIATE PROFESSOR
DEPARTMENT OF PEDIATRICS
CHILDREN'S HEALTH SERVICES RESEARCH
INDIANA UNIVERSITY SCHOOL OF MEDICINE
INDIANAPOLIS, IN 46202

ZENK, SHANNON N, RN, PHD
PROFESSOR
DEPARTMENT OF HEALTH SYSTEMS SCIENCE
COLLEGE OF NURSING
UNIVERSITY OF ILLINOIS AT CHICAGO
CHICAGO, IL 60612

MAIL REVIEWER(S)

DASANAYAKE, ANANDA P, BDS, PHD
PROFESSOR
DEPARTMENT OF EPIDEMIOLOGY
COLLEGE OF DENTISTRY
NEW YORK UNIVERSITY
NEW YORK, NY 10003

GIABBANELLI, PHILIPPE JOSEPH, PHD
ASSISTANT PROFESSOR
DEPARTMENT OF COMPUTER SCIENCE
NORTHERN ILLINOIS UNIVERSITY
DEKALB, IL 60115

SCIENTIFIC REVIEW OFFICER

WEIK, TASMEEN, DRPH, MPH
SCIENTIFIC REVIEW OFFICER
CENTER FOR SCIENTIFIC REVIEW
NATIONAL INSTITUTES OF HEALTH
BETHESDA, MD 20892

EXTRAMURAL SUPPORT ASSISTANT

SILLAH, RUGIATU
EXTRAMURAL SUPPORT ASSISTANT (INTERN)
CENTER FOR SCIENTIFIC REVIEW
NATIONAL INSTITTUES OF HEALTH
BETHESDA, MD 20892

* Temporary Member. For grant applications, temporary members may participate in the entire meeting or may review only selected applications as needed.

Consultants are required to absent themselves from the room during the review of any application if their presence would constitute or appear to constitute a conflict of interest.
